# Ablation alone is noninferior to radiotherapy plus ablation in the patients with early-stage hepatocellular carcinoma: a population-based study

**DOI:** 10.1038/s41598-024-51436-6

**Published:** 2024-01-10

**Authors:** Yusheng Guo, Hebing Chen, Jiayu Wan, Yanqiao Ren, Feihong Wu, Lei Chen, Tao Sun, Lian Yang, Chuansheng Zheng

**Affiliations:** 1 Department of Radiology, Union Hospital, Tongji Medical College, Huazhong University of Science and Technology, No. 1277 Jiefang Avenue, Wuhan, 430022 China; 2 Hubei Key Laboratory of Molecular Imaging, Wuhan, 430022 China

**Keywords:** Cancer, Computational biology and bioinformatics, Gastroenterology

## Abstract

Recently, the efficacy of two low-invasive treatments, ablation, and radiotherapy, has been fully compared for the patients with the early-stage hepatocellular carcinoma (HCC). However, the comparison between radiotherapy plus ablation and ablation alone has been less frequently reported. Data from the Surveillance, Epidemiology, and End Results (SEER) database were searched for early-stage HCC patients treated with ablation plus radiotherapy or ablation alone. The outcome measures were overall survival (OS) and cancer-specific survival (CSS). The propensity score matching (PSM) was used to reduce selection bias. We included 240 and 6619 patients in the radiotherapy plus ablation group and ablation group before the PSM. After PSM, 240 pairs of patients were included. The median OS (mOS) and median CSS (mCSS) of patients receiving ablation alone were longer than that of receiving radiotherapy plus ablation (mOS: 47 vs. 34 months, *P* = 0.019; mCSS: 77 vs. 40 months, *P* = 0.018, after PSM) before and after PSM. The multivariate analysis indicated that radiotherapy plus ablation independent risk factor for OS and CSS before PSM, but the significance disappeared after PSM. The detailed subgroup analyses indicated ablation alone brought more benefit in very early-stage HCC and older patients. In addition, we found different types of radiotherapy might lead to different outcomes when combined with ablation. In conclusion, ablation alone is noninferior to radiotherapy plus ablation in patients with early-stage HCC. The additional radiation prior to ablation may bring survival benefits in the patients with higher tumor stage. However, due to the risk of selection bias in that study, the results should be interpreted cautiously.

## Introduction

Hepatocellular carcinoma (HCC) is the sixth most common cancer and the fourth leading cause of cancer‐related deaths in the world^1^, accounting for about 75–85% of the incidence of all liver cancers^2^. For decades, radiotherapy did not play a substantial role in treating liver cancers due to the limited tolerance of the whole liver to radiation and complexity of tumor localization^3^. However, with the progress of imaging and radiation delivery, radiotherapy was proven to have potential efficacy across all stages of HCC^4^. The American Society for Radiation Oncology recently recommended external beam radiation therapy as the potential first-line treatment in patients with liver-confined HCC who were not suitable for curative therapy. In addition, radiotherapy was also recommended as a bridge treatment to liver transplant or before surgery in carefully selected patients^5^.

For the early-stage HCC, the potentially curative treatments include liver transplantation, partial hepatic resection, and ablation^6^. Ablation treatment mainly included radiofrequency ablation (RFA) which showed similar efficacy compared to surgical resection in patients with tumor size no more than 3 cm^7^. In addition to the similar efficacy, as a minimally invasive approach, ablation treatment could offer fewer complications, better safety and shorter hospital stay^8,9^. As a non-invasive treatment, the efficacy of radiotherapy mainly including stereotactic body radiation therapy (SBRT) in patients with early-stage HCC attracted more and more attention^10^. Hong et al. conducted a meta-analysis comparing SBRT vs. RFA in patients with small HCC and they reported that radiotherapy provided a higher local control ratio but a poorer prognosis than RFA^11^. Given that SBRT could serve as the supplementary therapeutic strategy when the lesion was attached to a vessel or located at subphrenic region, more and more researchers raised the point that these two types of treatment were not rivals but partners for the cure^12^. Considering the probability of incomplete RFA, the new strategy based on RFA plus radiotherapy was proposed by many researchers. They found RFA plus radiotherapy was safe and tolerated for patients^13–15^, meanwhile, patients who received RFA plus radiotherapy may have a lower local disease progression rate and better progression-free survival (PFS)^16^.

However, due to the relatively short follow-up periods or relatively small number of included patients in these studies. The aim of our study was to utilize the data from the Surveillance, Epidemiology, and End Results (SEER) database to compare the clinical outcomes of patients with early-stage HCC who received ablation alone and radiotherapy plus ablation. In addition, we investigated the efficacy of different types of radiotherapy plus ablation and the different sequences of radiotherapy plus ablation.

## Materials and methods

### Patient database

This population-based study was based on the US National Cancer Institute’s surveillance, epidemiology, and end results (SEER) database. The population-based data were obtained from Incidence-SEER 17 Regs Research Data (2000–2019) which covered up to 26.5% of the population in the USA. We used SEER*Stat statistical software, version 8.4.0.1 (National Cancer Institute, Bethesda, MD, USA) to collect information regarding demographics and diagnosis information. To be clear, the patients before 2004 have no information about TMN stages, so we selected patients with the year of diagnosis between 2004–2019.

### Patient selection

**Figure 1:**
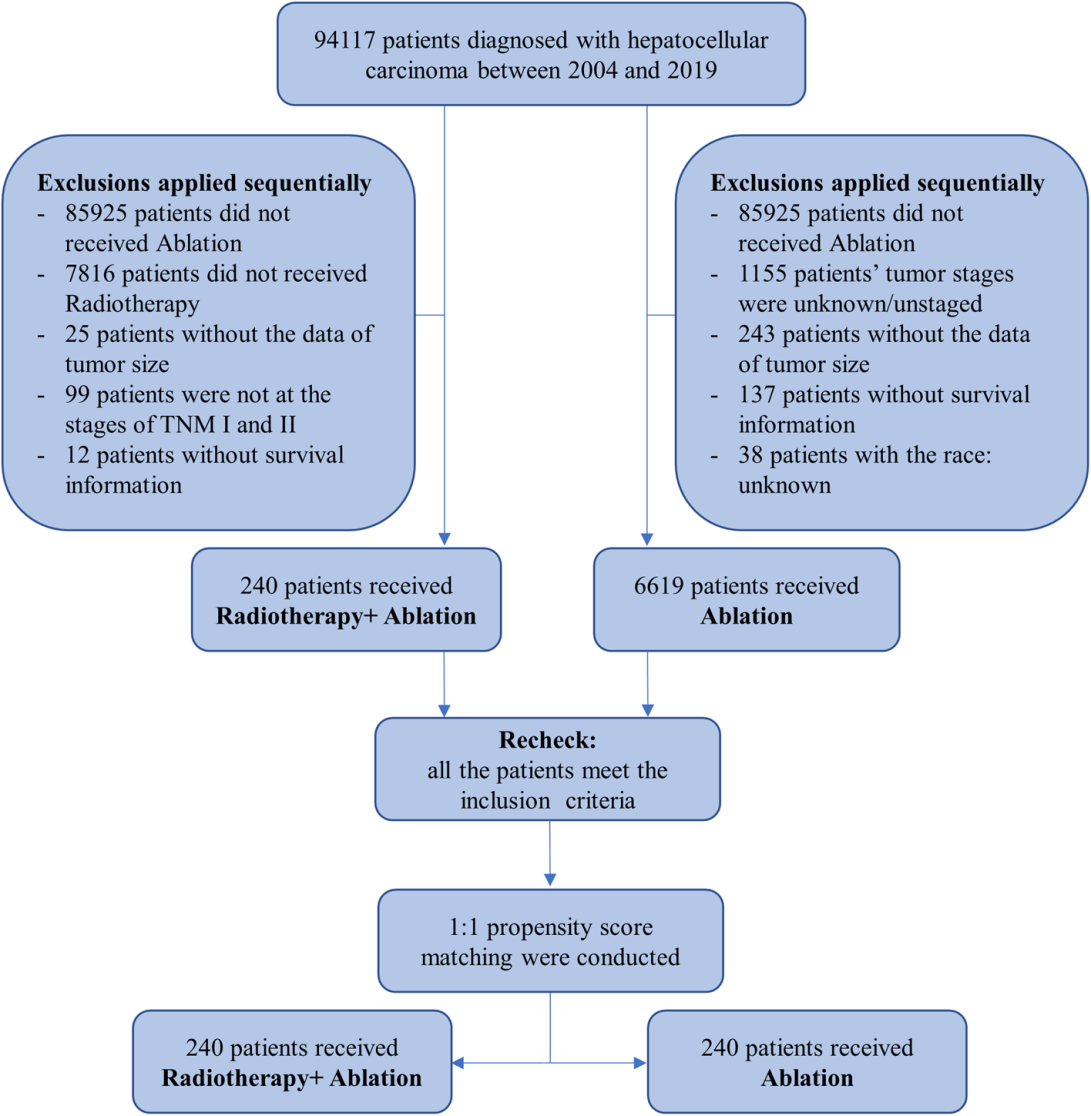
Flow chart of the patients included in the study.

According to the International Classification of Diseases for Oncology, third-edition histology codes, and the World Health Organization lymphoid classification (2008), we limited the patients who were histologically diagnosed with HCC (histology codes 8170/3–8175/3, site code C220.0). The patients with the following features were excluded: (1) patients did not receive the treatment of ablation; (2) patients’ tumor stages were unknown/unstaged; (3) patients were not at the stages of TNM I and II; (4) patients with loss of important information (tumor size, survival information, race). Figure 1 showed the flow of individuals through the screening process.

### Patient characteristics

For each case, we retrieved the characteristics of patients including age at diagnosis, year of diagnosis, gender, tumor stage (localized or regional), AJCC stage, tumor size, tumor number, race, marital status at diagnosis, the type of ablation, the presence of chemotherapy, survival months, survival months, and survival status (including vital status recode and SEER cause-specific death classification).

### Propensity score matching

Propensity score matching (PSM) analysis was used to reduce the selection bias due to baseline characteristics between the two groups. The variables including age at diagnosis, year of diagnosis, gender, tumor stage, AJCC stage, tumor size, tumor number, race, marital status at diagnosis, the type of ablation, and the presence of chemotherapy were used to perform PSM. One-to-one matching without replacement was applied, and the caliper value was 0.02.

### Statistical analysis

Age at diagnosis, year of diagnosis, and tumor size were converted from continuous variables into groups and analyzed as categorical variables. Categorical variables were analyzed using Pearson’s chi-square test or Fisher’s exact test. SEER cause-specific death classification (CSS) and overall survival (OS) curves between the radiotherapy plus ablation group and ablation group were made by the Kaplan–Meier method and the log-rank test was conducted to determine statistical significance. In addition, the log-rank Mantel-Cox test was applied for pairwise comparisons of survival data. Cox regression analysis was used for univariate analysis, in which variables with *P* value less than 0.05 in univariate analysis were added to multivariate analysis. All statistical analyses were conducted using SPSS 24.0 (IBM, Corp, NY, USA). *P*-value < 0.05 was considered statistically significant.

## Results

### Baseline characteristics

A total of 6859 patients with HCC were included in the study, with 240 and 6619 patients in the radiotherapy plus ablation group and ablation group before the PSM, respectively (Table 1). The age at diagnosis in the radiotherapy plus ablation group was older than that in the ablation group (*P* = 0.033). The years of diagnosis in the ablation group were earlier than that in the radiotherapy plus ablation group (*P* < 0.001). The patients who underwent radiotherapy plus ablation tended to have higher AJCC stage, larger tumor size, and more tumor numbers than whom received ablation treatment alone. Meanwhile, more patients in the ablation group also received the treatment of chemotherapy (69.90% vs. 32.08%, *P* < 0.001). In addition, the variable ethnicity between the two groups was unbalanced (*P* = 0.002) before the PSM. After the PSM, the *P*-values for all variables after were more than 0.1, which suggested that PSM effectively minimized the selection bias and imbalance between the two groups.

**Table 1:** The baseline characteristics of patients before and after PSM.

Characteristics	Before matching		*P* value	After matching		*P* value
	Radiotherapy + Ablation (n = 240)	Ablation (n = 6619)		Radiotherapy + Ablation (n = 240)	Ablation (n = 240)	
Age at diagnosis			0.033			0.171
≥ 65	129 (53.75%)	3095 (46.76%)		129 (53.75%)	114 (47.50%)	
< 65	111 (46.25%)	3524 (53.24%)		111 (46.25%)	126 (52.50%)	
Gender			0.079			0.494
Male	189 (78.75%)	4877 (73.68%)		189 (78.75%)	195 (81.25%)	
Female	51 (21.25%)	1742 (26.32%)		51 (21.25%)	45 (18.75%)	
Years of diagnosis			< 0.001			0.105
2004–2009	23 (9.58%)	1495 (22.59%)		23 (9.58%)	30 (12.50%)	
2010–2015	71 (29.58%)	2719 (41.08%)		71 (29.58%)	87 (36.25%)	
2016–2019	146 (60.84%)	2405 (36.33%)		146 (60.84%)	123 (51.25%)	
Tumor stage			< 0.001			0.74
Localized	181 (75.42%)	5822 (87.96%)		181 (75.42%)	197 (82.08%)	
Regional	59 (24.58%)	797 (12.04%)		59 (24.58%)	43 (14.92%)	
AJCC stage			< 0.001			0.113
I	136 (56.67%)	4833 (73.01%)		136 (56.67%)	153 (63.75%)	
II	104 (43.33%)	1786 (26.99%)		104 (43.33%)	87 (36.25%)	
Tumor size (cm)			< 0.001			0.649
No more than 3	114 (47.50%)	4113 (62.14%)		114 (47.50%)	123 (51.25%)	
3–5	104 (43.33%)	2196 (33.18%)		104 (43.33%)	99 (41.25%)	
Larger than 5	22 (9.17%)	310 (4.68%)		22 (9.17%)	18 (7.50%)	
Tumor number			< 0.001			0.977
1	179 (74.58%)	5688 (85.93%)		179 (74.58%)	180 (75.00%)	
2	49 (20.42%)	796 (12.03%)		49 (20.42%)	49 (20.42%)	
≥ 3	12 (5.00%)	135 (2.04%)		12 (5.00%)	11 (4.58%)	
Ethnicity			0.002			0.472
White	185 (77.08%)	4613 (69.90%)		185 (77.08%)	191 (79.58%)	
Black	28 (11.67%)	647 (9.77%)		28 (11.67%)	20 (8.33%)	
Other	27 (11.25%)	1359 (20.53%)		27 (11.25%)	29 (12.09%)	
Marital status			0.750			0.967
Married	128 (53.33%)	3598 (54.36%)		128 (53.33%)	127 (52.92%)	
Unmarried	100 (41.67%)	2754 (41.61%)		100 (41.67%)	102 (42.50%)	
Unknown	12 (5.00%)	267 (4.03%)		12 (5.00%)	11 (4.58%)	
Chemotherapy			< 0.001			0.371
Yes	77 (32.08%)	4627 (69.90%)		77 (32.08%)	68 (28.33%)	
No	163 (67.92%)	1992 (30.10%)		163 (67.92%)	172 (71.67%)	
Ablation			0.489			0.566
RFA	223 (92.92%)	6173 (93.26%)		223 (92.92%)	219 (91.25%)	
Cryoablation	2 (0.83%)	127 (1.92%)		2 (0.83%)	5 (2.08%)	
Laser ablation	2 (0.83%)	43 (0.65%)		2 (0.83%)	4 (1.67%)	
Alcohol ablation	13 (5.42%)	276 (4.17%)		13 (5.42%)	12 (5.00%)	

*AJCC* American Joint Committee on Cancer; *RFA* Radiofrequency ablation.

### Survival outcomes before and after PSM

**Figure 2:**
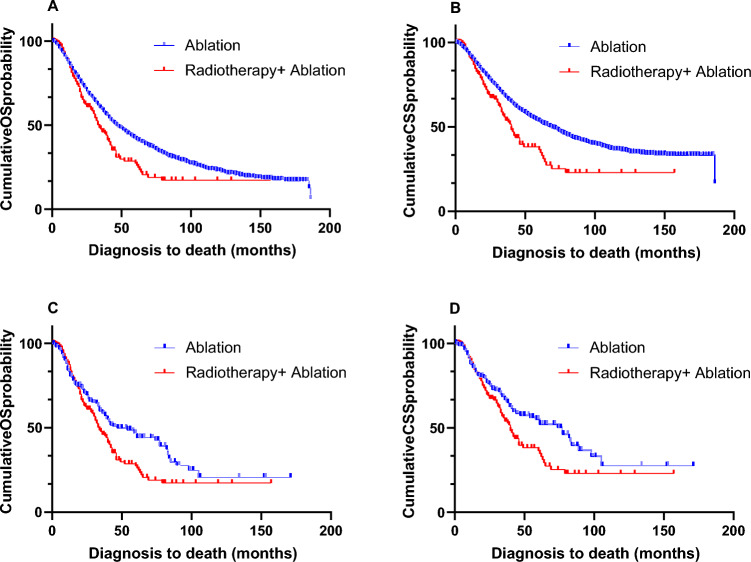
(**A**) Kaplan–Meier curve of OS before PSM; (**B**) Kaplan–Meier curve of CSS before PSM; (**C**) Kaplan–Meier curve of OS after PSM; (**D**) Kaplan–Meier curve of CSS after PSM.

Before the PSM, the median OS (mOS) in the ablation group was longer than mOS in the radiotherapy plus ablation group (47 vs. 34 months, *P* = 0.001, Fig. 2A). Similarly, the median CSS (mCSS) in the ablation group was longer than mCSS in the radiotherapy plus ablation group (69 vs. 40 months, *P* < 0.001, Fig. 2B). After the PSM, the mOS in the ablation group was still longer than that in the radiotherapy plus ablation group (47 vs. 34 months, *P* = 0.019, Fig. 2C), meanwhile, the mCSS achieved a similar result (77 vs. 40 months, *P* = 0.018, Fig. 2D).

**Figure 3:**
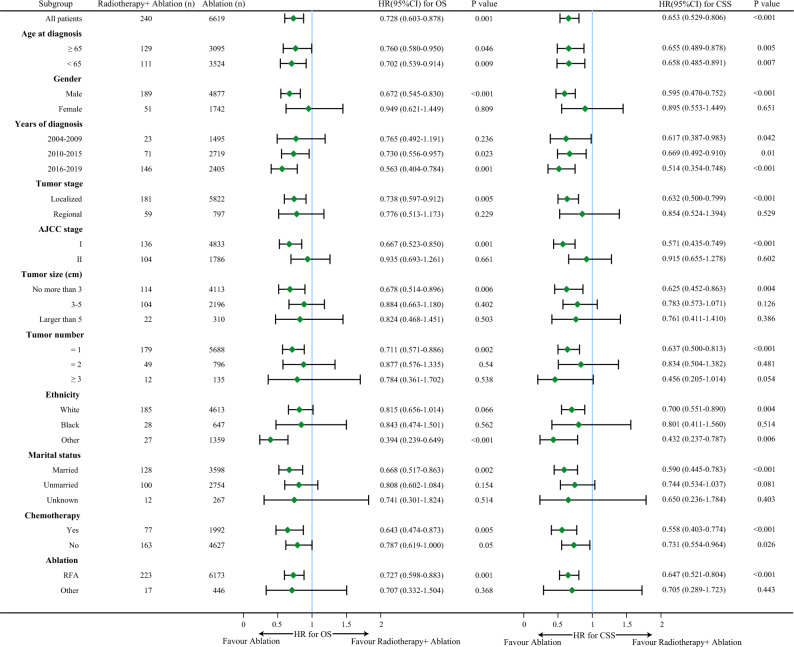
Forest plot of subgroup analysis before PSM.

**Figure 4:**
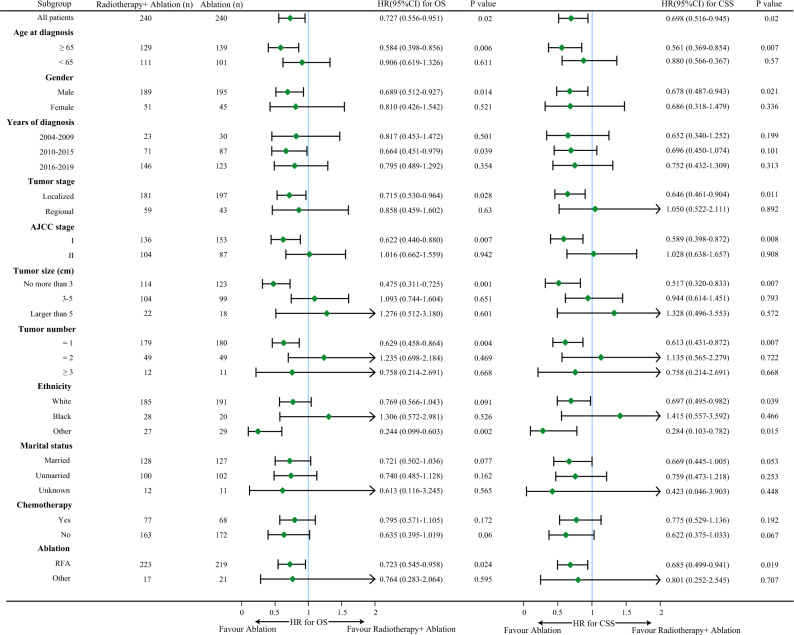
Forest plot of subgroup analysis after PSM.

Next, we conducted detailed subgroup analyses by forest plot according to the baseline characteristics of patients before and after PSM, the hazard ratios with 95% confidence intervals (CI) for the OS and CSS were shown in Figs. 3 and 4. Before the PSM, of note, we found that ablation alone was a favorable factor for OS and CSS in patients with earlier tumor stage (with tumor stage localized, with AJCC stage I, with tumor size no more than 3 cm, with single tumor), meanwhile, for patients with higher tumor stage, ablation alone tended to achieve similar efficacy compared with radiotherapy plus ablation (Fig. 3). After the PSM, we still found that ablation alone was a favorable factor for OS and CSS in patients with tumor stage localized (OS: HR = 0.715, 95%CI: 0.530–0.964, *P* = 0.028; CSS: HR = 0.646, 95%CI: 0.461–0.904, *P* = 0.011), patients with AJCC stage I (OS: HR = 0.622, 95%CI: 0.440–0.880, *P* = 0.007; CSS: HR = 0.589, 95%CI: 0.398–0.872, *P* = 0.008), patients with tumor size no more than 3 cm (OS: HR = 0.475, 95%CI: 0.311–0.725, *P* = 0.001; CSS: HR = 0.517, 95%CI: 0.320–0.833, *P* = 0.007), patients with single tumor (OS: HR = 0.629, 95%CI: 0.458–0.864, *P* = 0.004; CSS: HR = 0.613, 95%CI: 0.431–0.872, *P* = 0.007, Fig. 4).

### Cox regression analysis

Before the PSM, the univariable cox regression analysis indicated that the ablation alone was a favorable factor for OS (HR = 0.728, 95%CI: 0.603–0.878, *P* = 0.001) and CSS (HR = 0.653, 95%CI: 0.529–0.806, *P* < 0.001). To avoid over-fitting, the multivariable cox regression model contained only variables that had *P*-values < 0.05 in the univariable cox regression analysis. Similarly, multivariable analysis showed that ablation alone was a favorable factor for OS (HR = 0.811, 95%CI: 0.670–0.982, *P* = 0.031) and CSS (HR = 0.726, 95%CI: 0.587–0.898, *P* = 0.003, Table S1-S2).

After the PSM, the univariable cox regression analysis still showed that the ablation alone was a favorable factor for OS (HR = 0.727, 95%CI: 0.556–0.951, *P* = 0.020) and CSS (HR = 0.698, 95%CI: 0.516–0.945, *P* = 0.020). However, in the multivariable cox regression model, ablation alone was not an independent favorable factor for OS (HR = 0.779, 95%CI: 0.594–1.022, *P* = 0.071) and CSS (HR = 0.759, 95%CI: 0.559–1.030, *P* = 0.077, Tables 2, 3), although a modest trend was present.

**Table 2:** Univariable and multivariable cox regression analysis for OS after PSM.

Characteristics	Univariable analysis for OS		Multivariate analysis for OS	
	HR (95%CI)	*P* value	HR (95%CI)	*P* value
Age at diagnosis				
≥ 65	Reference			
< 65	1.093 (0.836, 1.429)	0.513		
Gender				
Male	Reference			
Female	0.774 (0.547, 1.095)	0.148		
Years of diagnosis				
2004–2009	Reference			
2010–2015	0.711 (0.500, 1.012)	0.058		
2016–2019	0.796 (0.534, 1.185)	0.261		
Tumor stage				
Localized	Reference		Reference	
Regional	1.421 (1.011, 1.998)	0.043	1.342 (0.951, 1.893)	0.094
AJCC stage				
I	Reference			
II	1.260 (0.957, 1.658)	0.099		
Tumor size (cm)				
No more than 3	Reference		Reference	
3–5	1.812 (1.365, 2.406)	< 0.001	1.746 (1.313, 2.323)	< 0.001
Larger than 5	2.476 (1.531, 4.004)	< 0.001	2.392 (1.473, 3.887)	< 0.001
Tumor number				
1	Reference			
2	1.343 (0.973, 1.853)	0.073		
≥ 3	1.584 (0.858, 2.923)	0.142		
Ethnicity				
White	Reference			
Black	1.139 (0.736, 1.762)	0.558		
Other	0.875 (0.578, 1.323)	0.526		
Marital status				
Married	Reference			
Unmarried	0.956 (0.726, 1.259)	0.749		
Unknown	0.788 (0.367, 1.690)	0.540		
Chemotherapy				
Yes	Reference			
No	0.842 (0.635, 1.116)	0.231		
Treatment				
Radiotherapy + Ablation	Reference		Reference	
Ablation	0.727 (0.556, 0.951)	0.020	0.779 (0.594, 1.022)	0.071

*OS* Overall Survival; *AJCC* American Joint Committee on Cancer; *RFA* Radiofrequency ablation.

**Table 3:** Univariable and multivariable Cox regression analysis for CSS after PSM.

Characteristics	Univariable analysis for CSS		Multivariate analysis for CSS	
	HR (95%CI)	*P* value	HR (95%CI)	*P* value
Age at diagnosis				
≥ 65	Reference			
< 65	0.974 (0.720, 1.317)	0.863		
Gender				
Male	Reference			
Female	0.684 (0.456, 1.027)	0.067		
Years of diagnosis				
2004–2009	Reference			
2010–2015	0.709 (0.478, 1.051)	0.087		
2016–2019	0.799 (0.510, 1.252)	0.328		
Tumor stage				
Localized	Reference			
Regional	1.431 (0.974, 2.102)	0.068		
AJCC stage				
I	Reference			
II	1.304 (0.958, 1.774)	0.092		
Tumor size (cm)				
No more than 3	Reference		Reference	
3–5	1.872 (1.359, 2.580)	< 0.001	1.820 (1.320, 2.511)	< 0.001
Larger than 5	2.789 (1.650, 4.715)	< 0.001	2.513 (1.477, 4.274)	0.001
Tumor number				
1	Reference			
2	1.119 (0.762, 1.644)	0.565		
≥ 3	1.960 (1.057, 3.637)	0.033		
Ethnicity				
White	Reference			
Black	1.118 (0.683, 1.829)	0.658		
Other	0.839 (0.524, 1.343)	0.464		
Marital status				
Married	Reference			
Unmarried	0.955 (0.701, 1.300)	0.769		
Unknown	0.717 (0.291, 1.766)	0.470		
Chemotherapy				
Yes	Reference		Reference	
No	0.718 (0.526, 0.979)	0.036	0.770 (0.563, 1.052)	0.100
Treatment				
Radiotherapy + Ablation	Reference		Reference	
Ablation	0.698 (0.516, 0.945)	0.020	0.759 (0.559, 1.030)	0.077

*CSS* Cancer-Specific Survival; *AJCC* American Joint Committee on Cancer; *RFA* Radiofrequency ablation.

### Types and sequences of radiotherapy analysis

**Figure 5:**
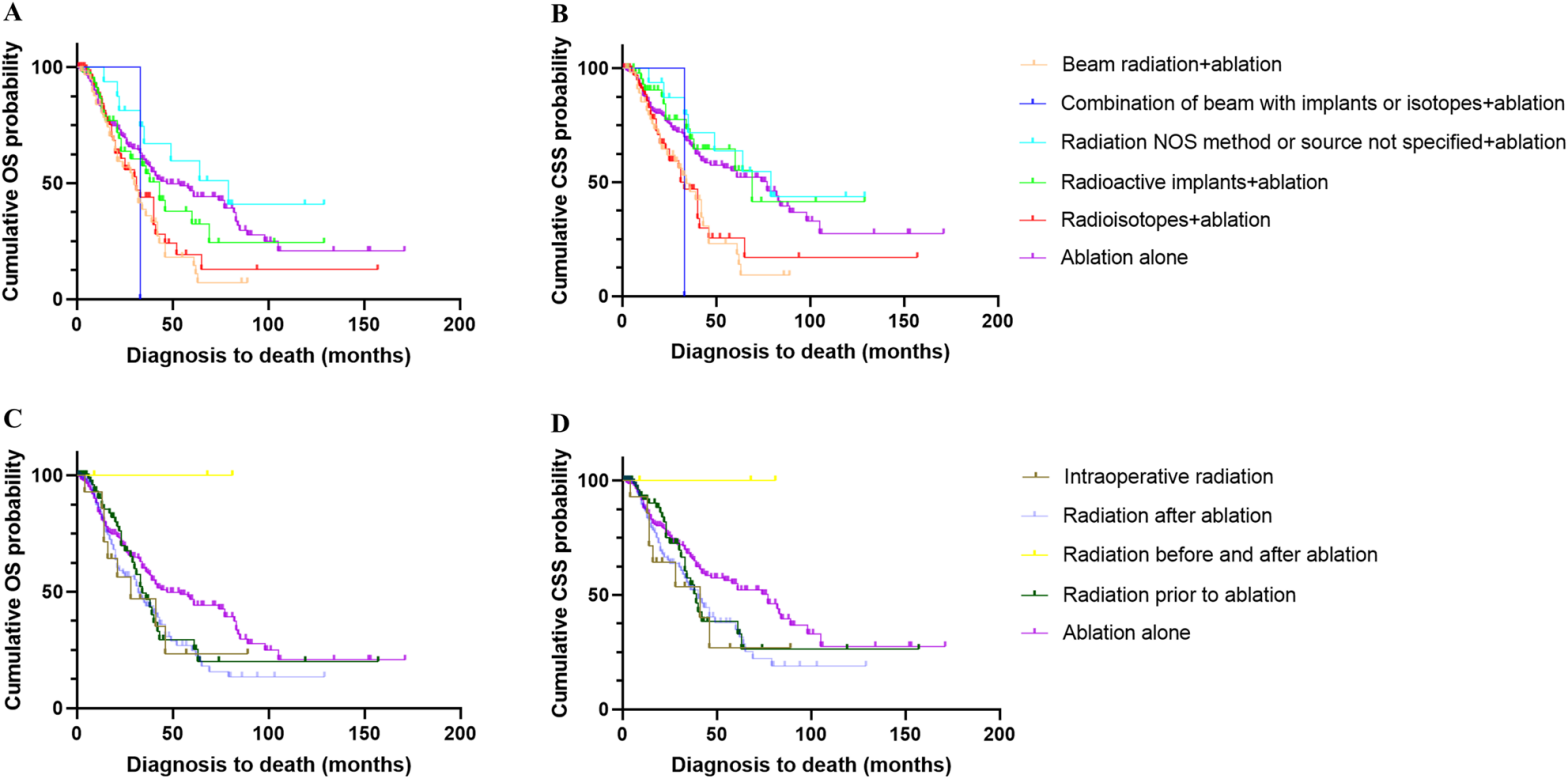
(**A**) Kaplan–Meier curve of OS in patients with different types of radiotherapy; (**B**) Kaplan–Meier curve of CSS in patients with different types of radiotherapy; (**C**) Kaplan–Meier curve of OS in patients with different sequences of radiotherapy; (**D**) Kaplan–Meier curve of CSS in patients with different sequences of radiotherapy.

The types and sequences of radiotherapy’s detailed information in radiotherapy plus ablation group (240 included patients) was listed in Table S3. We observed the types of radiotherapy may provide different survival outcomes when combine with ablation. The mOS in the patients with the radiotherapy type “Radiation, NOS method or source not specified” were significantly longer than patients with the radiotherapy type “Beam radiation” (79 vs. 30 months, *P* = 0.002, Fig. 5A) and type “Radioisotopes” (79 vs. 31 months, *P* = 0.013). The CSS achieved similar results (Fig. 5B), and it indicated that “Beam radiation” and “Radioisotopes” may impair the survival benefit brought by ablation (Table S3).

We compared the survival outcomes of patients with different sequences of radiotherapy. The result indicated that the mOS (32.0 vs. 47.0 months, *P* = 0.009, Fig. 5C) and mCSS (40.0 vs. 77.0 months, *P* = 0.012, Table S3, Fig. 5D) patients with “Radiation after ablation” were significantly shorter than patients who received ablation alone. “Radiation after ablation” means that radiation can be used as salvage therapy after a local recurrence. We found that patients with “Radiation after ablation” tended to have higher tumor stage at baseline compared to patients in the ablation alone group (regional: 26.1% vs 17.9%, *P* = 0.060, Table S4). The mOS (32.0 vs. 47.0 months, *P* = 0.335, Fig. 5C) and mCSS (39.0 vs. 77.0 months, *P* = 0.309, Fig. 5D) were similar in patients with “Radiation prior to ablation” and ablation alone group. The patients with “Radiation prior to ablation” have higher AJCC stage at baseline compared to patients in the ablation alone group (AJCC stage II: 49.4% vs 36.2%, *P* = 0.033, Table S5).

## Discussion

As the technique of radiotherapy evolves, the role of radiotherapy in the treatment of hepatocellular cancer has been explored. Previous studies have demonstrated the safety of SBRT plus ablation in early and even intermediate‐stage HCC^13–15^, therefore, we analyzed data from the publicly accessible SEER database to compare the efficacy of radiotherapy plus ablation and ablation alone in early-stage HCC.

Similar to surgical procedures^17^, radiotherapy given before ablation may help remove cancer cells that cannot be seen with Computed Tomography or Ultrasound, and convert tumors beyond indications for ablation into ablation-able tumors by shrinking the tumor. Meanwhile, due to the large size, irregular shape, and “heat sink effect” of the tumor^8^, radiotherapy given after ablation may help kill the residual HCC cells. However, we here yield a result less compatible with these hypotheses: we observed that ablation alone was noninferior to radiotherapy plus ablation in the patients with early-stage HCC from the perspective of overall outcomes, and ablation alone may gain more benefits in special subgroups. Combined with the results of subgroup analyses (Fig. 4), we found that ablation alone may bring more survival benefits to patients at very early-stage HCC with smaller tumor size and single tumor. One explanation could be that ablation alone was enough to kill most tumor cells in very early-stage HCC, additional radiotherapy may cause the unnecessary death of normal hepatocytes which may lead to potential radiation-induced liver injury (RILI). In addition, older age has been associated with higher rates of toxicity from radiotherapy^18^ and this could explain why we found that patients older than 65 were more likely to benefit from ablation alone.

The liver is a crucial organ and plays the most important role in various physiological functions such as bile acid circulation, glucose metabolism, lipid metabolism, protein metabolism and, immunity^19,20^. As a radiosensitive organ, hepatic toxicity from radiation therapy has been extensively reported^21^. The RILI can lead to the injury of hepatocytes and Kupffer cells, sinusoidal obstruction syndrome, and perivenular fibrosis^22^. The severity of RILI depends on the means of radiotherapy, the total exposure dose, the dose rate, and the physical area of exposure^19^. Therefore, we investigated the impact of different types and sequences of radiotherapy on survival outcomes. The results indicated that the different sequences of radiotherapy achieved similar results, and they both tended to be inferior to ablation alone (Fig. 5B). Notably, although the SEER database did not give the SBRT a special code number, many studies regarded “beam radiation” as SBRT therapy^23–25^. If we based on this hypothesis, we found that SBRT plus ablation might get the worst outcomes from the curves (Fig. 5A). However, a retrospective study reported that the safety and efficacy of SBRT plus ablation were similar to ablation alone in HCC patients at the stage of 0-B1^13^, therefore, we speculated that “beam radiation” may include other kinds of radiation therapy whose efficacy are worse than SBRT. Moreover, we needed to cleared that part of patients treated with combination therapy might use radiotherapy as a salvage treatment which might lead to selection bias in the population between two groups. Notably, when we compared the survival outcomes of patients with different sequences of radiotherapy, the patients with “Radiation after ablation” or “Radiation prior to ablation” tend to have higher tumor stage. Therefore, this additional radiotherapy may represent an imbalance in the population, either as salvage treatment after ablation or as treatment before ablation to reduce tumor burden. More specifically, this additional radiotherapy after ablation in the higher risk population did not bring additional survival benefits, while radiation prior to ablation bring additional early-stage survival benefits in the patients with higher AJCC stage (Fig. 5D).

The treatment of HCC has placed a severe economic burden on health care systems and each patient^26^. Parikh et al. reported that RFA and SBRT for early-stage hepatocellular carcinoma showed similar survival, 90-day hospitalization, or costs^27^ using the SEER-Medicare linked database. An earlier Markov modeling study reported that SBRT is the preferred salvage therapy for local progression after RFA in inoperable HCC from the point of cost-effectiveness. However, different from this study, we included patients with real and longer follow-up time (instead of model-simulated survival curves)^28^. Meanwhile, RFA has been reported to be less expensive compared with selective internal radiation therapy (SIRT)^29^. It is conceivable that no matter what type or sequence of radiotherapy is, the cost of radiotherapy plus ablation will be much higher than that of ablation alone. Consequently, ablation alone is still the better choice especially for patients with very early-stage HCC according to the results presented here.

The treatment decision to combine radiotherapy and ablation should be individualized. From a clinical practice perspective, tumors close to blood vessels or located in subphrenic area jeopardized local control of RFA^12^. The SBRT and SIRT were unaffected by the position of tumor, therefore, radiotherapy may still help improve local control of treatment. However, when tumor was far from a blood vessel or located in a non-subphrenic region, the clinician should consider with care whether additional radiotherapy could bring more survival benefits or not.

Our study has limitations. As with other SEER-based studies, the SEER database does not provide specific information about the type, dose, and timing of radiotherapy. Similarly, we did not get information about the postoperative complications and liver functions which were important to evaluate the safety of radiotherapy plus ablation. Like other retrospective studies, selection bias and confounding factors could have affected the results, but PSM, multivariate Cox regression models, and detailed subgroup analyses may help reduces these. Despite these statistical managements, this conclusion should be interpreted with caution.

## Conclusions

Ablation alone is noninferior to radiotherapy plus ablation in patients with early-stage HCC. The additional radiation prior to ablation may bring survival benefits in the patients with higher tumor stage. However, due to the risk of selection bias in that study, the results should be interpreted cautiously.

### Supplementary Information

Supplementary Information 1.

Supplementary Tables.

## Data Availability

Raw data used in analyses is available in supplementary materials and SEER database (http://seer.cancer.gov/seerstat).

## References

[1] Global, regional, and national life expectancy, all-cause mortality, and cause-specific mortality for 249 causes of death, 1980–2015: A systematic analysis for the Global Burden of Disease Study 2015. *Lancet (London, England)*. 2016;388(10053):1459–544. 10.1016/S0140-6736(16)31012-1 PMC5388903 27733281

[2] Bray F, Ferlay J, Soerjomataram I, Siegel RL, Torre LA, Jemal A (2018). Global cancer statistics 2018: GLOBOCAN estimates of incidence and mortality worldwide for 36 cancers in 185 countries. CA Cancer J. Clin..

[3] Kreidieh M, Zeidan YH, Shamseddine A (2019). The combination of stereotactic body radiation therapy and immunotherapy in primary liver tumors. J. Oncol..

[4] Kondo Y, Kimura O, Shimosegawa T (2015). Radiation therapy has been shown to be adaptable for various stages of hepatocellular carcinoma. World J. Gastroenterol..

[5] Apisarnthanarax S, Barry A, Cao M, Czito B, DeMatteo R, Drinane M (2022). External beam radiation therapy for primary liver cancers: An ASTRO clinical practice guideline. Pract. Radiat. Oncol..

[6] Reig M, Forner A, Rimola J, Ferrer-Fàbrega J, Burrel M, Garcia-Criado Á (2022). BCLC strategy for prognosis prediction and treatment recommendation: The 2022 update. J. Hepatol..

[7] Xu Z, Xie H, Zhou L, Chen X, Zheng S (2019). The combination strategy of transarterial chemoembolization and radiofrequency ablation or microwave ablation against hepatocellular carcinoma. Anal. Cell. Pathol. (Amsterdam)..

[8] Guo Y, Ren Y, Dong X, Kan X, Zheng C (2022). An overview of hepatocellular carcinoma after insufficient radiofrequency ablation. J. Hepatocell. Carcinoma.

[9] Cucchetti A, Piscaglia F, Cescon M, Colecchia A, Ercolani G, Bolondi L (2013). Cost-effectiveness of hepatic resection versus percutaneous radiofrequency ablation for early hepatocellular carcinoma. J. Hepatol..

[10] Wahl DR, Stenmark MH, Tao Y, Pollom EL, Caoili EM, Lawrence TS (2016). Outcomes after stereotactic body radiotherapy or radiofrequency ablation for hepatocellular carcinoma. J. Clin. Oncol..

[11] Hong J, Cao L, Xie H, Liu Y, Yu J, Zheng S (2021). Stereotactic body radiation therapy versus radiofrequency ablation in patients with small hepatocellular carcinoma: A systematic review and meta-analysis. Hepatobiliary Surg. Nutr..

[12] Kim N, Seong J (2022). Stereotactic body radiation therapy and radiofrequency ablation in patients with hepatocellular carcinoma: Not a rival but a partner for the cure. Hepatobiliary Surg. Nutr..

[13] Wang F, Numata K, Takeda A, Ogushi K, Fukuda H, Hara K (2021). Safety and efficacy study: Short-term application of radiofrequency ablation and stereotactic body radiotherapy for Barcelona Clinical Liver Cancer stage 0–B1 hepatocellular carcinoma. PLoS ONE.

[14] Wang F, Numata K, Takeda A, Ogushi K, Fukuda H, Nihonmatsu H (2021). Optimal application of stereotactic body radiotherapy and radiofrequency ablation treatment for different multifocal hepatocellular carcinoma lesions in patients with Barcelona Clinic Liver Cancer stage A4–B1: A pilot study. BMC Cancer.

[15] Fu Y, Xi M, Pan Y, Chen J, Wang J, Liu S (2020). Stereotactic body radiotherapy as a salvage therapy after incomplete radiofrequency ablation for hepatocellular carcinoma: A retrospective cohort study. J. Oncol..

[16] Pan YX, Xi M, Fu YZ, Hu DD, Wang JC, Liu SL (2019). Stereotactic body radiotherapy as a salvage therapy after incomplete radiofrequency ablation for hepatocellular carcinoma: A retrospective propensity score matching study. Cancers (Basel)..

[17] Gillen S, Schuster T, Meyer Zum Büschenfelde C, Friess H, Kleeff J (2010). Preoperative/neoadjuvant therapy in pancreatic cancer: A systematic review and meta-analysis of response and resection percentages. PLoS Med..

[18] Zeng C, Wen W, Morgans AK, Pao W, Shu XO, Zheng W (2015). Disparities by race, age, and sex in the improvement of survival for major cancers: Results from the national cancer institute surveillance, epidemiology, and end results (SEER) program in the United States, 1990 to 2010. JAMA Oncol..

[19] Zhu W, Zhang X, Yu M, Lin B, Yu C (2021). Radiation-induced liver injury and hepatocyte senescence. Cell Death Discov..

[20] Lai KK, Kolippakkam D, Beretta L (2008). Comprehensive and quantitative proteome profiling of the mouse liver and plasma. Hepatology (Baltimore, Md)..

[21] Li J, Xu J, Lu Y, Qiu L, Xu W, Lu B (2016). MASM, a matrine derivative, offers radioprotection by modulating lethal total-body irradiation-induced multiple signaling pathways in wistar rats. Molecules (Basel, Switzerland)..

[22] Takamatsu S, Kozaka K, Kobayashi S, Yoneda N, Yoshida K, Inoue D (2018). Pathology and images of radiation-induced hepatitis: A review article. Jpn. J. Radiol..

[23] Oladeru OT, Miccio JA, Yang J, Xue Y, Ryu S, Stessin AM (2016). Conformal external beam radiation or selective internal radiation therapy—a comparison of treatment outcomes for hepatocellular carcinoma. J. Gastrointest. Oncol..

[24] Li M, Xu X, Qin Y, Zhang P, Shen C, Xia Q (2021). Radiofrequency ablation vs. stereotactic body radiotherapy for stage IA non-small cell lung cancer in nonsurgical patients. J. Cancer.

[25] Yang ZL, Sun XQ, Tang YH, Xiong PY, Xu L (2022). Comparison of stereotactic body radiation therapy with hepatic resection and radiofrequency ablation as initial treatment in patients with early-stage hepatocellular carcinoma. Front. Oncol..

[26] Scheau C, Badarau IA, Caruntu C, Mihai GL, Didilescu AC, Constantin C (2019). Capsaicin: Effects on the pathogenesis of hepatocellular carcinoma. Molecules (Basel, Switzerland)..

[27] Parikh ND, Marshall VD, Green M, Lawrence TS, Razumilava N, Owen D (2018). Effectiveness and cost of radiofrequency ablation and stereotactic body radiotherapy for treatment of early-stage hepatocellular carcinoma: An analysis of SEER-medicare. J. Med. Imaging Radiat. Oncol..

[28] Pollom EL, Lee K, Durkee BY, Grade M, Mokhtari DA, Wahl DR (2017). Cost-effectiveness of stereotactic body radiation therapy versus radiofrequency ablation for hepatocellular carcinoma: A markov modeling study. Radiology..

[29] Ray CE, Battaglia C, Libby AM, Prochazka A, Xu S, Funaki B (2012). Interventional radiologic treatment of hepatocellular carcinoma-a cost analysis from the payer perspective. J. Vasc. Intervent Radiol. JVIR.

