# Supplementary material for: Ablation alone is noninferior to radiotherapy plus ablation in the patients with early-stage hepatocellular carcinoma: a population-based study
Source: Sci Rep. 2024 Jan 10;14:1030. doi: 10.1038/s41598-024-51436-6 (PMC10781784; doi:10.1038/s41598-024-51436-6)
Supplement: Supplementary file 2 — Supplementary Tables. [file 41598_2024_51436_MOESM2_ESM.docx]

Table S1 Univariable and multivariable Cox regression analysis for OS before PSM

| Characteristics | Univariable analysis for OS | | Multivariate analysis for OS | |
| --- | --- | --- | --- | --- |
|  | HR (95%CI) | *P* value | HR (95%CI) | *P* value |
| Age at diagnosis |  |  |  |  |
| ≥65 | Reference |  | Reference |  |
| <65 | 0.764 (0.715,0.817) | <0.001 | 0.743 (0.694,0.797) | <0.001 |
| Gender |  |  |  |  |
| Male | Reference |  |  |  |
| Female | 0.956 (0.886,1.031) | 0.245 |  |  |
| Years of diagnosis |  |  |  |  |
| 2004-2009 | Reference |  | Reference |  |
| 2010-2015 | 0.760 (0.706, 0.819) | <0.001 | 0.785 (0.728, 0.847) | <0.001 |
| 2016-2019 | 0.609 (0.548, 0.677) | <0.001 | 0.605 (0.543, 0.674) | <0.001 |
| Tumor stage |  |  |  |  |
| Localized | Reference |  | Reference |  |
| Regional | 1.337 (1.213, 1.473) | <0.001 | 1.162 (1.040, 1.299) | 0.008 |
| AJCC stage |  |  |  |  |
| I | Reference |  | Reference |  |
| II | 1.324 (1.233, 1.421) | <0.001 | 1.239 (1.142, 1.346) | <0.001 |
| Tumor size (cm) |  |  |  |  |
| No more than 3 | Reference |  | Reference |  |
| 3-5 | 1.542 (1.440, 1.652) | <0.001 | 1.461 (1.362, 1.568) | <0.001 |
| Larger than 5 | 1.786 (1.559, 2.046) | <0.001 | 1.695 (1.474, 1.950) | <0.001 |
| Tumor number |  |  |  |  |
| 1 | Reference |  | Reference |  |
| 2 | 1.186 (1.075, 1.308) | 0.001 | 1.078 (0.975, 1.192) | 0.143 |
| ≥3 | 1.592 (1.286, 1.971) | <0.001 | 1.404 (1.132, 1.742) | 0.002 |
| Ethnicity |  |  |  |  |
| White | Reference |  | Reference |  |
| Black | 0.893 (0.798, 0.999) | 0.049 | 0.927 (0.828, 1.038) | 0.187 |
| Other | 0.675 (0.619, 0.735) | <0.001 | 0.649 (0.595, 0.709) | <0.001 |
| Marital status |  |  |  |  |
| Married | Reference |  | Reference |  |
| Unmarried | 1.130 (1.056, 1.209) | <0.001 | 1.146 (1.070, 1.227) | <0.001 |
| Unknown | 1.062 (0.892, 1.263) | 0.500 | 1.114 (0.936, 1.325) | 0.226 |
| Chemotherapy |  |  |  |  |
| Yes | Reference |  | Reference |  |
| No | 0.951 (0.885, 1.021) | 0.162 | 1.058 (0.983, 1.138) | 0.133 |
| Treatment |  |  |  |  |
| Radiotherapy+ Ablation | Reference |  | Reference |  |
| Ablation | 0.728 (0.603, 0.878) | 0.001 | 0.811 (0.670, 0.982) | 0.031 |

Note. OS: Overall Survival; AJCC: American Joint Committee on Cancer; RFA: Radiofrequency ablation.

Table S2 Univariable and multivariable Cox regression analysis for CSS before PSM

| Characteristics | Univariable analysis for CSS | | Multivariate analysis for CSS | |
| --- | --- | --- | --- | --- |
|  | HR (95%CI) | *P* value | HR (95%CI) | *P* value |
| Age at diagnosis |  |  |  |  |
| ≥65 | Reference |  | Reference |  |
| <65 | 0.754 (0.698, 0.816) | 0.001 | 0.730 (0.674, 0.790) | <0.001 |
| Gender |  |  |  |  |
| Male | Reference |  |  |  |
| Female | 0.952 (0.871, 1.041) | 0.284 |  |  |
| Years of diagnosis |  |  |  |  |
| 2004-2009 | Reference |  | Reference |  |
| 2010-2015 | 0.732 (0.671, 0.798) | <0.001 | 0.772 (0.706, 0.843) | <0.001 |
| 2016-2019 | 0.601 (0.531, 0.680) | <0.001 | 0.610 (0.538, 0.693) | <0.001 |
| Tumor stage |  |  |  |  |
| Localized | Reference |  | Reference |  |
| Regional | 1.451 (1.299, 1.622) | <0.001 | 1.168 (1.029, 1.326) | 0.016 |
| AJCC stage |  |  |  |  |
| I | Reference |  | Reference |  |
| II | 1.478 (1.362, 1.605) | <0.001 | 1.365 (1.241, 1.501) | <0.001 |
| Tumor size (cm) |  |  |  |  |
| No more than 3 | Reference |  | Reference |  |
| 3-5 | 1.669 (1.539, 1.810) | <0.001 | 1.552 (1.428, 1.686) | <0.001 |
| Larger than 5 | 1.996 (1.707, 2.332) | <0.001 | 1.866 (1.589, 2.191) | <0.001 |
| Tumor number |  |  |  |  |
| 1 | Reference |  |  |  |
| 2 | 1.055 (0.936, 1.190) | 0.382 |  |  |
| ≥3 | 1.260 (0.954, 1.664) | 0.103 |  |  |
| Ethnicity |  |  |  |  |
| White | Reference |  | Reference |  |
| Black | 0.887 (0.776, 1.014) | 0.079 | 0.934 (0.817, 1.068) | 0.316 |
| Other | 0.699 (0.631, 0.773) | <0.001 | 0.661 (0.597, 0.732) | <0.001 |
| Marital status |  |  |  |  |
| Married | Reference |  | Reference |  |
| Unmarried | 1.005 (0.815, 1.239) | 0.963 |  |  |
| Unknown | 1.119 (0.906, 1.381) | 0.296 |  |  |
| Chemotherapy |  |  |  |  |
| Yes | Reference |  | Reference |  |
| No | 0.871 (0.802, 0.945) | 0.001 | 0.993 (0.912, 1.080) | 0.862 |
| Treatment |  |  |  |  |
| Radiotherapy+ Ablation | Reference |  | Reference |  |
| Ablation | 0.653 (0.529, 0.806) | <0.001 | 0.726 (0.587, 0.898) | 0.003 |

Note. CSS: Cancer-Specific Survival; AJCC: American Joint Committee on Cancer; RFA: Radiofrequency ablation.

Table S3 Types or sequences of radiotherapy

| Types or sequences of radiotherapy | Radiotherapy+ Ablation  (n=240) | Median OS  (95CI%) | Median CSS  (95CI%) |
| --- | --- | --- | --- |
| **The types of radiotherapy** |  |  |  |
| Beam radiation | 61 | 30.0 (22.5-37.5) | 34.0 (23.1-44.2) |
| Combination of beam with implants or isotopes | 1 | / | / |
| Radiation, NOS method or source not specified | 16 | 79.0 (34.4-123.6) | 79.0 (35.3-122.7) |
| Radioactive implants | 48 | 47.0 (33.0-61.0) | 69.0 (50.1-87.9) |
| Radioisotopes | 114 | 31.0 (21.3-40.7) | 33.0 (26.3-40.0) |
| **The sequences of radiotherapy** |  |  |  |
| Intraoperative radiation | 14 | 28.0 (1.7-54.3) | 41.0 (8.7-73.3) |
| Radiation after ablation | 138 | 32.0 (26.5-37.5) | 40.0 (30.6-49.4) |
| Radiation before and after ablation | 3 | / | / |
| Radiation prior to ablation | 85 | 34.0 (26.6-41.4) | 39.0 (32.2-45.8) |
| / | Ablation  (n=240) | Median OS  (95CI%) | Median CSS  (95CI%) |
| / | 240 | 47.0 (33.0-61.0) | 77.0 (54.9-99.1) |

Note. OS: Overall Survival; CSS: Cancer-Specific Survival; AJCC: American Joint Committee on Cancer; RFA: Radiofrequency ablation.

Table S4 The comparison of characteristics between Ablation alone group and Radiation after ablation group

| Characteristics | Ablation alone | Radiation after ablation | *P* value |
| --- | --- | --- | --- |
| n | 240 | 138 |  |
| Age at diagnosis, n (%) |  |  | 0.590 |
| <65 | 101 (42.1%) | 62 (44.9%) |  |
| ≥65 | 139 (57.9%) | 76 (55.1%) |  |
| Gender, n (%) |  |  | 0.175 |
| female | 45 (18.8%) | 34 (24.6%) |  |
| male | 195 (81.2%) | 104 (75.4%) |  |
| Years of diagnosis, median (IQR) | 2016 (2012, 2018) | 2017 (2013.2, 2018) | 0.003 |
| Tumor stage, n (%) |  |  | 0.060 |
| Localized | 197 (82.1%) | 102 (73.9%) |  |
| Regional | 43 (17.9%) | 36 (26.1%) |  |
| AJCC stage, n (%) |  |  | 0.676 |
| I | 153 (63.7%) | 85 (61.6%) |  |
| II | 87 (36.2%) | 53 (38.4%) |  |
| Tumor size, n (%) |  |  | 0.691 |
| No more than 3 | 123 (51.2%) | 77 (55.8%) |  |
| 3-5 | 99 (41.2%) | 52 (37.7%) |  |
| Larger than 5 | 18 (7.5%) | 9 (6.5%) |  |
| Tumor number, n (%) |  |  | 0.863 |
| 1 | 180 (75%) | 100 (72.5%) |  |
| 2 | 49 (20.4%) | 31 (22.5%) |  |
| ≥3 | 11 (4.6%) | 7 (5.1%) |  |
| Ethnicity, n (%) |  |  | 0.804 |
| white | 191 (79.6%) | 109 (79%) |  |
| black | 20 (8.3%) | 14 (10.1%) |  |
| other | 29 (12.1%) | 15 (10.9%) |  |
| Marital status, n (%) |  |  | 0.589 |
| Married | 127 (52.9%) | 76 (55.1%) |  |
| Unmarried | 102 (42.5%) | 53 (38.4%) |  |
| Unknown | 11 (4.6%) | 9 (6.5%) |  |
| Ablation, n (%) |  |  | 0.577 |
| Cryoablation | 5 (2.1%) | 1 (0.7%) |  |
| Laser ablation | 4 (1.7%) | 1 (0.7%) |  |
| Alcohol ablation | 12 (5%) | 9 (6.5%) |  |
| Radiofrequency ablation | 219 (91.2%) | 127 (92%) |  |
| Chemotherapy, n (%) |  |  | 0.244 |
| yes | 68 (28.3%) | 47 (34.1%) |  |
| no | 172 (71.7%) | 91 (65.9%) |  |

Note. AJCC: American Joint Committee on Cancer

Table S5 The comparison of characteristics between Ablation alone group and Radiation prior to ablation group

| Characteristics | Ablation alone | Radiation prior to ablation | *P* value |
| --- | --- | --- | --- |
| n | 240 | 85 |  |
| Age at diagnosis, n (%) |  |  | 0.174 |
| <65 | 101 (42.1%) | 43 (50.6%) |  |
| ≥65 | 139 (57.9%) | 42 (49.4%) |  |
| Gender, n (%) |  |  | 0.639 |
| female | 45 (18.8%) | 14 (16.5%) |  |
| male | 195 (81.2%) | 71 (83.5%) |  |
| Years of diagnosis, median (IQR) | 2016 (2012, 2018) | 2017 (2014, 2018) | 0.002 |
| Tumor stage, n (%) |  |  | 0.176 |
| Localized | 197 (82.1%) | 64 (75.3%) |  |
| Regional | 43 (17.9%) | 21 (24.7%) |  |
| AJCC stage, n (%) |  |  | 0.033 |
| I | 153 (63.7%) | 43 (50.6%) |  |
| II | 87 (36.2%) | 42 (49.4%) |  |
| Tumor size, n (%) |  |  | 0.062 |
| No more than 3 | 123 (51.2%) | 31 (36.5%) |  |
| 3-5 | 99 (41.2%) | 45 (52.9%) |  |
| Larger than 5 | 18 (7.5%) | 9 (10.6%) |  |
| Tumor number, n (%) |  |  | 0.911 |
| 1 | 180 (75%) | 65 (76.5%) |  |
| 2 | 49 (20.4%) | 17 (20%) |  |
| ≥3 | 11 (4.6%) | 3 (3.5%) |  |
| Ethnicity, n (%) |  |  | 0.171 |
| white | 191 (79.6%) | 61 (71.8%) |  |
| black | 20 (8.3%) | 13 (15.3%) |  |
| other | 29 (12.1%) | 11 (12.9%) |  |
| Marital status, n (%) |  |  | 0.497 |
| Married | 127 (52.9%) | 42 (49.4%) |  |
| Unmarried | 102 (42.5%) | 41 (48.2%) |  |
| Unknown | 11 (4.6%) | 2 (2.4%) |  |
| Ablation, n (%) |  |  | 0.938 |
| Cryoablation | 5 (2.1%) | 1 (1.2%) |  |
| Laser ablation | 4 (1.7%) | 1 (1.2%) |  |
| Alcohol ablation | 12 (5%) | 4 (4.7%) |  |
| Radiofrequency ablation | 219 (91.2%) | 79 (92.9%) |  |
| Chemotherapy, n (%) |  |  | 0.822 |
| yes | 68 (28.3%) | 23 (27.1%) |  |
| no | 172 (71.7%) | 62 (72.9%) |  |

Note. AJCC: American Joint Committee on Cancer
